# Determining the Optimal Restricted Driving Zone Using Genetic Algorithm in a Smart City

**DOI:** 10.3390/s20082276

**Published:** 2020-04-16

**Authors:** Tony Jan, Pegah Azami, Saeid Iranmanesh, Omid Ameri Sianaki, Shiva Hajiebrahimi

**Affiliations:** 1 School of IT and Engineering, Melbourne Institute of Technology, Sydney, NSW 2000, Australia; tjan@mit.edu.au; 2 Computer Science, Laurentian University, Sudbury, ON P3E 2C6, Canada; pegah.azami2003@gmail.com; 3 Business School, Victoria University, Melbourne, VIC 3000, Australia; omid.amerisianaki@vu.edu.au; 4 Information Systems Engineering, Gina Cody School of Engineering and Computer Science, Concordia University, Montreal, QC H3G 1M8, Canada; s_hajieb@encs.concordia.ca

**Keywords:** restricted driving zone, genetic algorithm, smart city, air pollution, traffic management

## Abstract

Traffic control is one of the most challenging issues in metropolitan cities with growing populations and increased travel demands. Poor traffic control can result in traffic congestion and air pollution that can lead to health issues such as respiratory problems, asthma, allergies, anxiety, and stress. The traffic congestion can also result in travel delays and potential obstruction of emergency services. One of the most well-known traffic control methods is to restrict and control the access of private vehicles in predetermined regions of the city. The aim is to control the traffic load in order to maximize the citizen satisfaction given limited resources. The selection of restricted traffic regions remains a challenge because a large restricted area can reduce traffic load but with reduced citizen satisfaction as their mobility will be limited. On the other hand, a small restricted area may improve citizen satisfaction but with a reduced impact on traffic congestion or air pollution. The optimization of the restricted zone is a dynamic multi-regression problem that may require an intelligent trade-off. This paper proposes Optimal Restricted Driving Zone (ORDZ) using the Genetic Algorithm to select appropriate restricted traffic zones that can optimally control the traffic congestion and air pollution that will result in improved citizen satisfaction. ORDZ uses an augmented genetic algorithm and determinant theory to randomly generate different foursquare zones. This fitness function considers a trade-off between traffic load and citizen satisfaction. Our simulation studies show that ORDZ outperforms the current well-known methods in terms of a combined metric that considers the least traffic load and the most enhanced citizen satisfaction with over 30.6% improvements to some of the comparable methods.

## 1. Introduction

Intelligent Transportation System (ITS) plays an important role in managing traffic in the large cities to reduce traffic congestion and air pollution, and to satisfy the citizens. The needs of such a system become more evident in a highly populated large city with growing population and increased travel demands. The increased travel demands and limited road capacity can cause traffic jam resulting in the dissatisfaction of the citizens. The increased travel time can additionally cause the emission of carbon monoxide [1]. Consequently, stress, anxiety [2], and more serious health problems such as respiratory, digestive, blood, metabolic, ophthalmological, circulatory, musculoskeletal diseases can occur [3].

The current methods of traffic control can be categorized into four types, namely (I) classic (traditional) methods that change the patterns of urban trips by encouraging citizens to use public transportation rather than private transportation. Although this method has significant impacts on reducing air pollution and traffic congestion, the lack of public infrastructure and low quality of services may propel people to opt private vehicles in a developing country where the public infrastructure may be poor [4], (II) smart traffic lights that can control the signal timing based on the traffic condition in real time i.e., number of vehicles on the road and the length of the queues. One of the benefits of smart traffic lights is the reduced delay time in intersections and the improved support for emergency vehicles [5]. Despite its benefits, the smart traffic method will need overly expensive equipment in its implementation [6], (III) modern technology methods can take advantage of hybrid electric vehicles on reduction of air pollution index and fossil fuel [7], but this method has high maintenance cost, (IV) limited traffic zone methods can restrict mobility of private vehicles based on some criteria i.e., road pricing, restrict traffic zone, and odd-even traffic restriction. In this paper, we focus on the limited traffic zone methods that are cost-effective for developing countries. These methods are used in many cities with high population such as Beijing and Tehran. For example, an odd and even zone method can give access to vehicles to move in a zone based on its license plate number and the corresponding day of the week [8]. The odd and even zone approach was implemented in Beijing during 2008 Olympic Games. During this period, the air pollution index was reduced by 30% [9]. The range of these zones; however, was not optimal and was only specific to the Olympic Games requirements [10]. Defining the restricted zone is a challenge in statistical optimization with several factors to consider—reducing traffic congestion, reducing air pollution, and satisfying the citizens. The cost-effectiveness solution such as the above can be a very attractive option for many developing cities.

Based on the aforementioned problem statement and the lack of an efficient method to control traffic in a large city with poor public infrastructure is problematic in many cities. These cities are mostly from the developing countries in Asia and the other continents where there is only limited public infrastructure but rapidly growing as the regional business center. They need a resolution on controlling their traffic to sustain their business activities as well as to reduce air pollution. In this regard, we examine the limited traffic zone in detail as it provides a viable and cost-effective solution for the developing countries. To further improve the traditional limited traffic zone approaches, this paper incorporates an innovative genetic algorithm in optimizing the restricted drive zones [11]. This paper proposes an innovative Optimal Restricted Driving Zone (ORDZ) method based on Genetic Algorithm (GA) to reduce the traffic load, air pollution and improving citizen satisfaction ratings [12].

The proposed ORDZ uses genetic algorithms with a multi-objective fitness function that satisfies the required criteria. Genetic algorithm is a random and non-deterministic method for finding solutions in optimization and search problems [13]. This algorithm is a type of evolutionary algorithm so that it solves complex problems by using natural mechanisms such as inheritance (chromosomes and genes) and mutations. The genetic algorithm provides a possible interim solution until it reaches the optimal solution using the fitness function. The genetic algorithm determines the traffic zone with a geometric shape, and in this paper, an innovative solution is suggested on chromosome formulation in the genetic algorithm that uses mathematical objects called determinant for achieving an optimal learning with regularization.

The contributions of this paper are as follows:

- An innovative technique called Optimal Restricted Driving Zone (ORDZ) is introduced that intelligently determines restricted driving zones using a machine learning technique. RDZ reduces traffic load and air pollution, and increase citizens satisfaction who wish to travel by their own vehicle. All the aforementioned objectives are formulated into a single multi-objective function that constitute an evolutionary algorithm called genetic algorithm. While the previous works [8,9] determine restrict zone empirically, ORDZ generates a possible solution iteratively until it reaches the optimal solution (with each episode creating a viable solution). This approach has a significant advantage in dynamic traffic conditions as shown in the following sections.
- In our simulation, ORDZ is compared against the other well-known methods including: the Restricted Traffic Zone (RTZ) [8], the Odd-Even Zone (OEZ) [8] and the optimal cordon-based network congestion based on pricing (OCP) [14]. We compare our work against the other well-known empirical approaches. The performance of each method is evaluated with two metrics, traffic load and citizen satisfaction rate. The results show that ORDZ has 23.81% less traffic load than OCP. Also, ORDZ has 22.35% increase in citizen satisfaction than RTZ. We also compare both metrics together as a trade-off and a complete solution. The results show that ORDZ performs 30.6% better than the random modeling, empirical methods, RTZ, OEZ and OCP in terms of traffic load and citizen satisfaction rates.

The rest of this paper is organized as follows. Section 2 describes the related work. Section 3 describe the underlying traffic control systems. Section 4 introduces the ORDZ method. Section 5 evaluates ORDZ against the other methods, and finally, Section 6 concludes the paper.

## 2. Related Work

The traffic management systems can be categorized into four types namely; (i) traditional methods of changing the urban trip patterns, (ii) smart traffic light methods in which signal timing adaptively changes based on the traffic conditions (queues), (iii) modern technology with vehicles using clean fuel, and (iv) restricting traffic zone for accessibility.

In the following, we will discuss the advantages and limitations of each category given the conditions of largely populated cities in Asia with limited public transport infrastructures. The availability of public transport changes the modeling landscape; therefore, we limit our study to the case of Tehran which is the largest city in Iran. The proposal of this paper may be relevant to other cities in developing countries in Asia and other continents.

### 2.1. Public Transportation

The public transportation system is a part of many of the traffic control systems. In [15], Anwar et al. made comparison between public and private transport systems. They argued that the public transport system has the benefits of reducing air pollution, fuel consumption and travel times. In [16], Le et al. investigated that one of the ways to solve traffic congestion problems is to use public transportation. However, in many developing cities such as Dhaka, the capital of Bangladesh, 56% of private car owners state that they do not wish to use the public transportation because of the poor quality of service. Hence, people in the countries with poor public infrastructure may have more inclination to use private vehicles. Meanwhile, Salarvandian et al. [8], concluded that 50% of air pollution in big cities such as Tehran is caused by the private vehicles.

### 2.2. Smart Traffic Lights Mmethods

Smart traffic light methods control the signal timing adaptively based on the current traffic conditions. In [17], Geng et al. proposed a new method to reducing the travel time in emergency using a fuzzy model in smart cities. In this modeling, the traffic light time intervals are changed adaptively to provide priority service to emergency vehicles and services as follows: (1) change the status of traffic lights, (2) change of speeds, (3) release the lines for emergency vehicles, (4) allow using pre-booked lines, and (5) using redirection protocols. In [5], Alam et al. presented a method of calculating traffic flow using a fuzzy logic approach. In this method, two electromagnetic sensors are placed on each side of the road to gather information on the traffic congestion. The advantage of this method is that the intersection capacity is increased with a reduced delay. Some consider it the equivalent of the traffic control by the human police officers at the crossroads. In [8,18], using a fuzzy logic control and image processing techniques, they were able to optimize the traffic signal time for both low and high traffic conditions. Hence, they were able to implement real life scenario to control the traffic flow. The limitations of such an approach are the high cost of sensor devices and poor decision outcomes under adversely weather conditions. These limitations turn out to be too costly for some developing countries.

### 2.3. Modern Technology Method

In [19], Muhid et al. proposed a method of examining the effects of hybrid electric vehicles on reduction of air pollution index. The electric vehicles consume less energy as compared to the gasoline vehicles. The advantage of replacing electric cars is that its effective in reducing air pollution over a long term. Also, Soret et al. [7] measured the emissions by the hybrid and electric vehicles, which according to their research, reduces the air pollution index by 11%.

### 2.4. Restricted Driving Zone Methods

Limited traffic zone method can be a cost-effective solution for a developing country. The limited traffic zone schemes are categorized as follows; (*i*) road pricing (*ii*) restrict traffic zone (*iii*) odd-even traffic restriction. In [14], Zhang et al. proposed a method in determining an optimal traffic zone in Shanghai city center. In their work, two performance metrics are considered such as social welfare and generalized travel cost. They used genetic algorithm, theory of graph and direct grid search method to determine the optimal traffic zone. However, this scheme did not consider the traffic flow which is an important parameter in modeling the traffic pattern. In this paper, we endeavor to consider the traffic flow in determining the traffic zones.

In [8], the authors considered the capital city of Iran, Tehran for managing the extensive vehicles in this city. In this city, two traffic zones are created in the city center that include: Restricted Traffic Zone (RTZ) using pass permits and Odd-Even Zone (OEZ) based on the license plate numbers. These restricted driving zones are determined by characteristics such as the covered area (km${}^{2}$), population density and environmental characteristics such as the size of commercial and residential areas in the region. The status of each traffic zone is defined by an indicator, which addresses the volume-to-capacity ratio. The volume is the number of vehicles that pass through a point within an hour. In terms of capacity, there is a distribution in several vehicles that can cross a road line in an appropriate time. In RTZ, it is known as the main traffic zone and the access to this zone is limited to between 6:30 a.m. and 5:00 p.m. on weekdays. For OEZ, they considered both constant and variable approaches. In the constant approach, the specific days are allowed for odd plate number and the other days for even plate numbers. In this way, the control system may not have any predictions about traffic load requirements. In the variable pattern, the traffic quota for vehicles on the weekdays is determined by the last digit on the license plate for a fixed period, and the restriction pattern changes for the next cyclic periods. The benefits of odd/even traffic restriction are to reduce the personal vehicle usage. The disadvantage is that car owners with an odd licenses plate number are allowed to pass zone in fewer days than even licenses plate number.

### 2.5. Discussion

To date, researchers have conducted many studies to reduce traffic load including classic methods, smart traffic lights, limited traffic zone and modern technology (See Table 1). Classic (traditional) method requires an adequate public infrastructure to motivate people to use public transportation which can be a challenge in a developing country. Smart traffic lights are proposed in order to reduce traffic flow at the intersections; however, the resource required for sensor deployment and management can be overwhelming in a developing country with limited resources. Modern technology also is very costly for the people to purchase the vehicles of advanced technologies. Hence, the approach with traffic zone restriction is considered more efficient and appropriate for developing countries including Iran and other Asian nations. In the following, we consider the traffic controlling system with advanced zone restriction methods.

## 3. System Description

**Figure 1:**
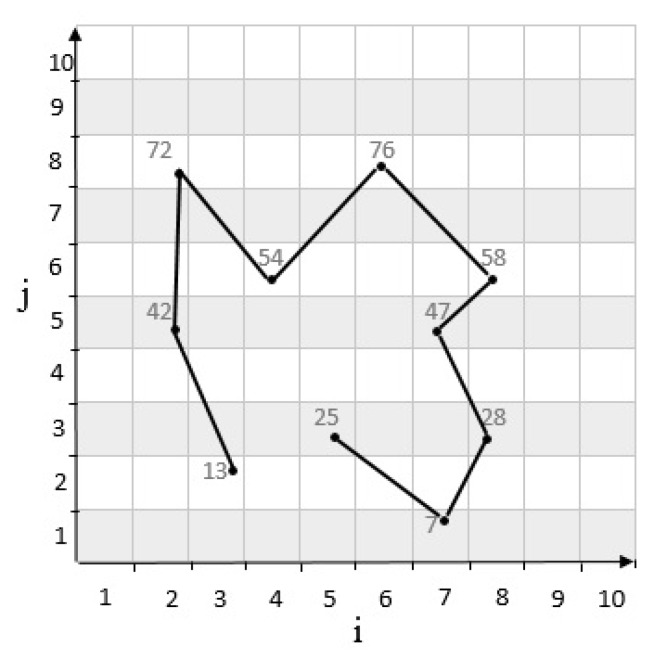
Movement pattern of $u_{1}$ in grid cell with m×m dimension.

Let us consider a city with m×m grid cell using mask. In the city, vehicles are movable and will be shown by $U=\left(u_{1},\cdots ,u_{i},\cdots ,u_{r}\right\}$ with $u_{i}$ representing *i*-th vehicle. Moreover, each vehicle is placed on one of the grid cells at time *t*. The grid cells are represented by $\left(c_{11},\cdots ,c_{ij},\cdots ,c_{mm}\right\}$. Here, $c_{ij}$ is a cell in *i*-th row and *j*-th column with $i,j\in \left(1,2,\cdots ,m\right\}$. In addition, each cell has mapped to a number as follows:

(1) $$c_{ij}=\left(j-1\right)\times m+i,$$

where *j* is the vertical coordinate and *i* is the horizontal coordinate of a cell. Also, m is the dimension of the cells. In other words, the Equation (1) represents each cell as a number in consideration of its location on the grid cell. For example, considering a grid cell with m=5 and the cell which located at row 4 and column 2 is $\left(c_{42}\right)$. According to Equation (1), $c_{42}$ is calculated as follows: $c_{42}=\left(2-1\right)\times 5+4=9$. The movement of vehicle $\left(u_{i}\right)$ on a grid cell is according to the movement pattern as ${MP}_{u_{i}}$ which is shown as a regular pair $\left(c_{ij},t\right)$. In other words, $c_{ij}$ shows the cell number which the vehicle is located at time *t* as a discrete time $\left(t=\left(1,2,3,\cdots ,T\right\}\right)$. Moreover, dynamic movement pattern is considered for vehicles which are able to start new patterns many times. For instance, taxi movement pattern is constantly changing as the demand from the passengers change. According to Figure 1, a grid cell with m=10 h (100 cells) is considered which $u_{1}$ is supposed to pass cells through $\left(c_{32},c_{25},c_{28},c_{46},c_{68},c_{86},c_{75},c_{83},c_{71},c_{53}\right\}$ in 10 time units. Therefore, the vehicle’s movement pattern is:

$${MP}_{u_{1}}=\left(\left(13,1\right),\left(42,2\right),\left(72,3\right),\left(54,4\right),\left(76,5\right),\left(58,6\right),\left(47,7\right),\left(28,8\right),\left(7,9\right),\left(25,10\right)\right\}$$

Table 2 contains the notations that used in the system description.

### Problem Definition

Traffic control and management is one of the most challenging issues in urban modeling and controlling. Some urban areas have a larger volume of traffic due to the demand of its inhabitant in work and commercial activities. Therefore, some of these urban zones require an intelligent traffic zone control to manage traffic load and air pollution. If a small traffic zone is selected, citizen satisfaction will increase but it will not reduce the traffic load. On the other hand, if an extensive traffic zone is selected, it may reduce the traffic load but increase in citizen dissatisfaction. An intelligent traffic zone control model is required to provide a suitable trade-off and an optimal selection of the traffic zones.

***Scenario 1***: Assume that the traffic zone is determined but air pollution index is not decreased. This case indicates that the zone was not significant enough and did not have effect on the reduction of air pollution. Therefore, a more extended zone is required to effectively reduce air pollution. The determination of optimal limited traffic zone has a significant impact on reducing air pollution.

***Scenario 2***: Assume that an extensive traffic limited zone is selected, and the traffic and air pollution indexes are reduced; however, at the cost of increased dissatisfaction of the citizens. Therefore, finding an optimal traffic zone is a challenge that leads to citizen satisfaction and reduced traffic and air pollution.

## 4. Proposed Method: Optimal Restricted Driving Zone (ORDZ)

In this section, a new method named Optimal Restricted Driving Zone (ORDZ) is proposed to define the traffic zone better and achieve acceptable trade-off between citizen satisfaction and congestion reduction. In the proposed model, the extensibility of the zone is determined by the information including the movement patterns of the vehicles as well as the other information. In this approach, each cell group that are traversed by the vehicles will form a geometric shape for “the limited traffic zone”. Therefore, each zone is assessed based on the predicted model of vehicle movements to reduce traffic load but increase the citizen satisfaction rating as the vehicle routes will have minimum traffic load possible with predictive modeling. The selected zone in such an approach will have more flexibility to control the traffic load in dynamic situations including rush hours.

### 4.1. Initial Plan

In this paper, ORDZ uses a population-based system in which the genetic algorithm is used for population evaluation and natural selection process. Due to the random selection of cells, ORDZ has higher chance of establishing the optimal zone that attain reduced traffic load and citizen satisfaction. In addition, the cell groups are organized as geometric shapes to define the movement patterns which is used to determine the traffic zone. This zone is optimized by constraining the traffic with mathematical determinants. In this regard, the genetic algorithm is based on Darwinian evolutionary theory which states that only species from a population of generations with the best features thrives on and those who do not have this feature will be gradually eliminated. Also, the new generation will have better capabilities than its predecessor. This algorithm is a type of evolutionary algorithm that uses natural mechanisms such as inheritance (chromosomes and genes) and mutation to solving complex problems. The chromosome consists of a set of possible responses, which consists of a multiplicity of genes that represent the variables. The problem-solving process in the genetic algorithm is that the algorithm randomly generates a set of solutions for the given problem, which forms the primary population. Each member of the population is displayed with a chromosome and each chromosome is coded according to the nature of the problem. Then, the intersection operator is applied to the chromosomes and children are produced by exchanging genes of the parent chromosomes. Given the present population of parents and children, we make a jump that changes the gene or genes of the chromosomes randomly to create a new child. The mutation operator prevents the local optimal occurrence. In this way, the population is composed of parent chromosomes and children’s chromosomes that are formed in combination with mutation. The current population will be evaluated by competency function and the most suitable chromosome is selected as the next-generation parent. This process continues until an optimal solution is achieved in a complex problem.

### 4.2. Chromosome Formulation

**Figure 2:**
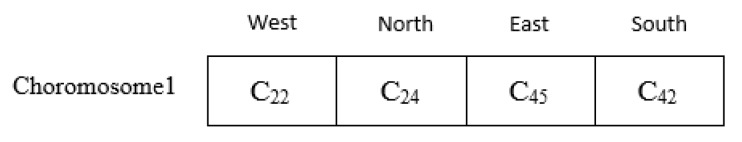
Format of encoded chromosome.

**Figure 3:**
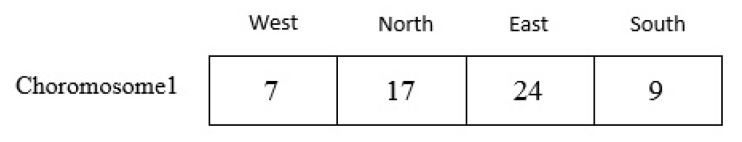
Example of encoded chromosome.

In this paper, the optimal traffic zone is determined by the genetic algorithm, in order to display the response to the problem by the use of chromosomes. Each chromosome consists of a set of cells that are randomly selected and must form a scope; therefore, we apply restrictions on the selection of cells, so that each cell is one selected direction of either North, South, East and West in the grid cells. Hence, we will have a chromosome with length of 4, each chromosome gene contains the coordinates of the $c_{ij}$ cell. For example, Figure 2 shows a coding chromosome that contains $c_{22},c_{24},c_{45},c_{42}$ cells and the value is demonstrated in Figure 3.

### 4.3. Constraints Satisfaction

The vehicle’s movement on grid cell begins from a cell and then meets a group of cells which can be organized as the geometric shapes to represent a traffic zone. Therefore, in order to determine the above zone, there should be restrictions on cell selection. Therefore, the cell’s location is considered to be one of the directions of the North, South, East and West. For example, vehicle’s movement begins from a cell in the west and continues to the next cell on the east side of the grid. Then two sequential cells are connected linearly, representing that the third cell cannot be selected on this line. The location of the third cell where the vehicle can meet is also located in the upper or lower area of the line. In other words, the third cell is placed in one of the directions of the north or south of the grid cell, and this process continues until it forms a zone. Hence, using the determinant method approximates the location of a cell relative to a line. Determinants in mathematical concepts are referred to as the functions that assign each square matrix to a number.

Hence, the determinants are used to determine the special values of a matrix or, in other words, a linear mapping. The determinants of R for the three cells $c_{i_{1}j_{1}},c_{i_{2}j_{2}},c_{i_{3}j_{3}}$ with coordinates, $\left(i_{1},j_{1}\right),\;\left(i_{2},j_{2}\right),\;\left(i_{3},j_{3}\right)$ is calculated as follows:

(2) $$R=\left|\begin{array}{cc} \left(i_{2}-i_{1}\right) & \left(i_{3}-i_{1}\right)\\ \left(j_{2}-j_{1}\right) & \left(j_{3}-j_{1}\right) \end{array}\right|=\;\left[\left(i_{2}-i_{1}\right)\times \left(j_{3}-j_{1}\right)-\left(i_{3}-i_{1}\right)\times \left(j_{2}-j_{1}\right)\right]$$

If the result of determinants is positive, the third cell’s location is placed on top or left of the line. If the result of determinants is negative, the third cell’s location is placed on the bottom or right of the line; and if the result of determinants is zero, the third cell is on the line. There are three possible states in consideration of the determinant values.

**Figure 4:**
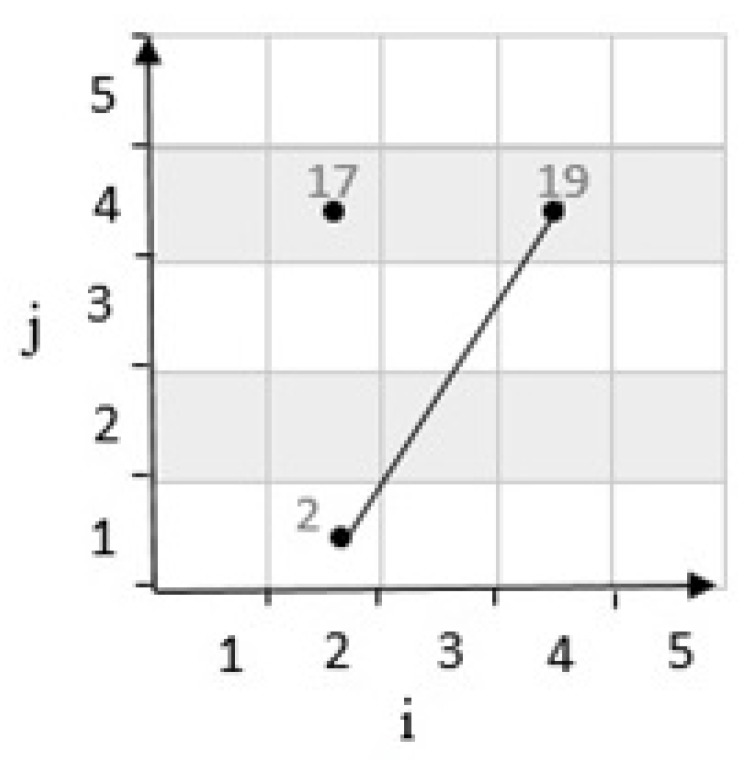
Position of a point with positive determinant.

**Figure 5:**
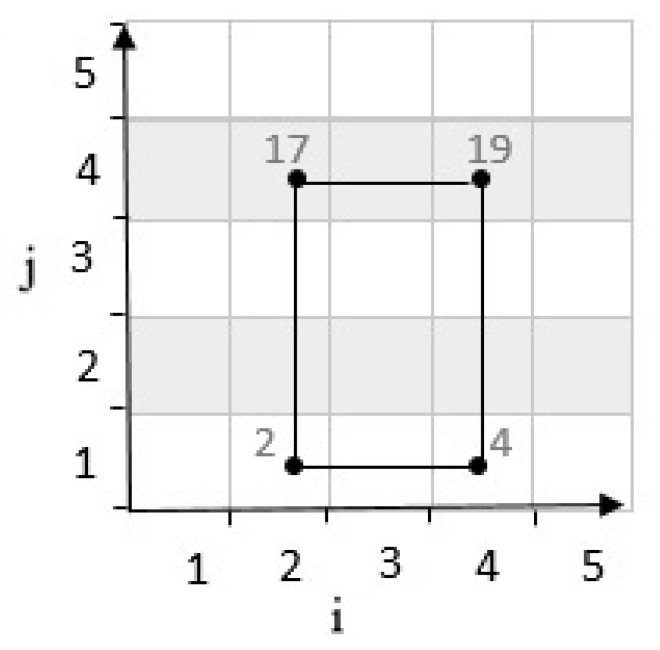
Position of fourth point with positive determinant.

**First state:** If the result of the determinant $\left(R\right)$ is positive, it means that the third cell is located on top of the line. For example, considering cells $c_{21},c_{44}$ with coordinates $\left(2,1\right)$ and $\left(4,4\right)\;$ on a grid cell with m=5 in Figure 4. According to Equation (1), $c_{21},c_{44}$ are mapped to numbers 2 and 19. These two cells are connected by a line. Then, by calculating the determinants, the position of the third cell $c_{24}$ with the coordinates $\left(2,4\right)$ which is mapped to 17, and it is determined as follows:

After determining the location of the third cell, the location of the fourth cell where the vehicle can meet is in front of the third cell, as described in Figure 5. In other words, if the third cell is in the north of the grid cell, the fourth cell is located in the south as shown in Figure 5.

**Figure 6:**
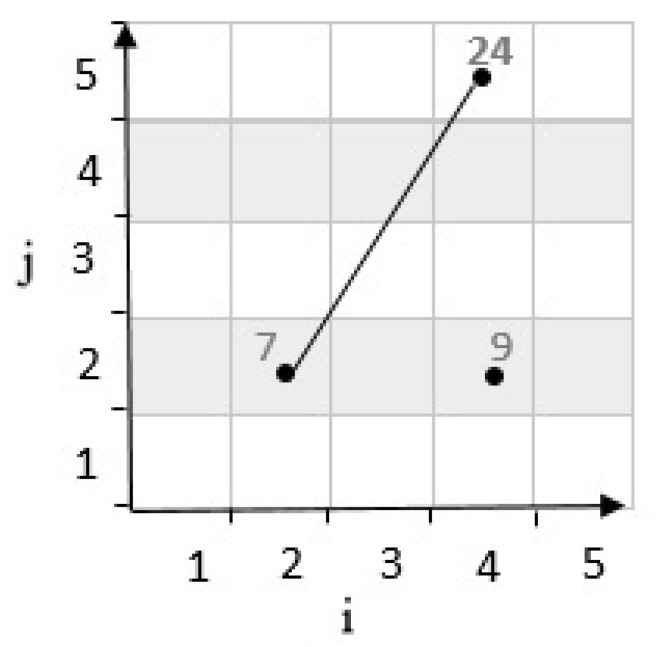
Position of a point with negative determinant.

**Second state:** If the result of determinants $\left(R\right)$ is negative, the third cell’s location is placed on the bottom of the line. For example, in Figure 6, considering cells $c_{22},c_{45}$ have coordinates $\left(2,2\right)$ and $\left(4,5\right)$ in a grid cell with m=5. According to Equation (1), $c_{22},c_{45}$ are mapped to numbers 7 and 24. These two cells are connected by a line. Then, by calculating the determinants, the position of the third cell $c_{42}$ with the coordinates $\left(4,2\right)$ which is mapped to 9, and it is determined as follows:

(3) $$R=\left|\begin{array}{cc} \left(i_{2}-i_{1}\right) & \left(i_{3}-i_{1}\right)\\ \left(j_{2}-j_{1}\right) & \left(j_{3}-j_{1}\right) \end{array}\right|=\left|\begin{array}{cc} \left(4-2\right) & \left(4-2\right)\\ \left(5-2\right) & \left(2-2\right) \end{array}\right|=0-6=-6<0$$

**Figure 7:**
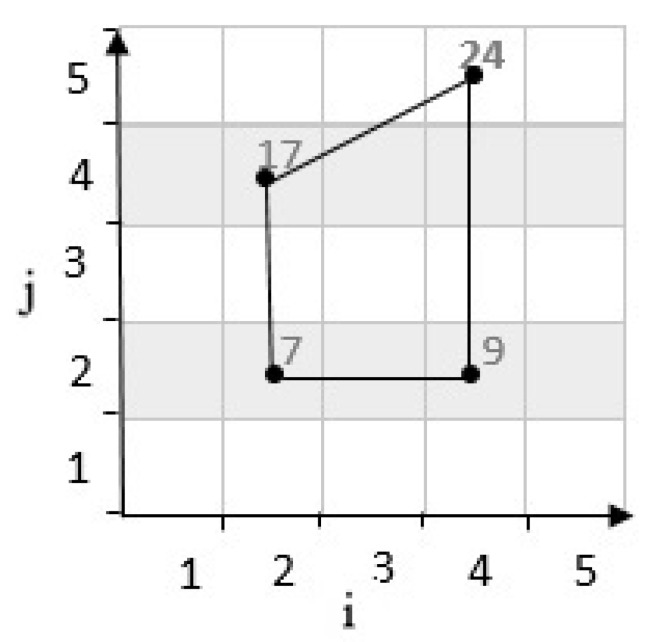
Position of fourth point with negative determinant.

Therefore, after determining the third cell’s location, the location of the fourth cell is chosen to form a zone. As shown in Figure 7, the fourth cell’s location is in the direction of the north of the grid cell.

**Figure 8:**
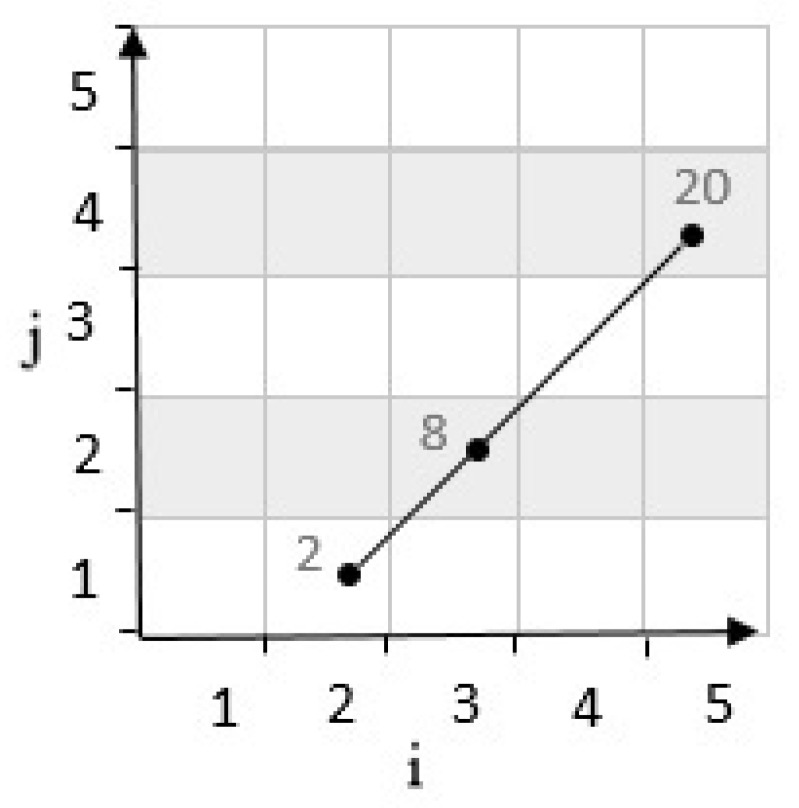
Position of a point with neutral determinant.

**Third state**: If the result of the determinant $\left(R\right)$ is zero, the third cell is placed on the line. So, it does not form a zone. For example, in Figure 8, considering cells $c_{21},c_{54}$ have coordinates $\left(2,1\right)$ and $\left(5,4\right)$ in a grid cell with m=5. According to Equation (1), $c_{21},c_{54}$ are mapped to numbers 2 and 20. These two cells are connected by a line. Then, by calculating the determinants, the position of the third cell $c_{32}$ with the coordinates $\left(3,2\right)$ which is mapped to 8, and it is determined as follows:

(4) $$R=\left|\begin{array}{cc} \left(i_{2}-i_{1}\right) & \left(i_{3}-i_{1}\right)\\ \left(j_{2}-j_{1}\right) & \left(j_{3}-j_{1}\right) \end{array}\right|=\left|\begin{array}{cc} \left(5-2\right) & \left(3-2\right)\\ \left(4-1\right) & \left(2-1\right) \end{array}\right|=3-3=0$$

### 4.4. Initial Population

**Figure 9:**
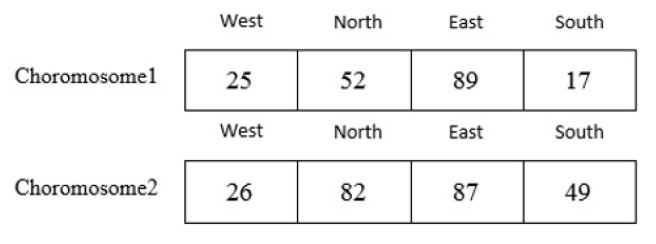
Primary population sample.

**Figure 10:**
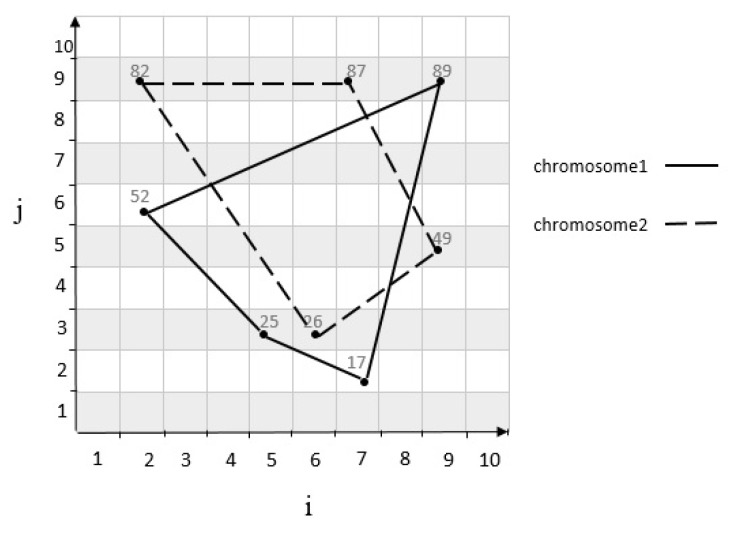
Scope of parent chromosomes.

The genetic algorithm generates an initial population μ in search of the optimal traffic zone. The initial population is equal to the number of cells randomly selected to meet the demand of their traffic control. Also, the limitations must be applied to the selected cells. Thus, the parent chromosomes are selected from the initial population μ. For example, suppose we have a grid cell with m=10 as shown in Figure 9, two sets of parent chromosomes are selected with length 4 from the initial population μ. A chromosome 1 genes contains following cells: $c_{53},c_{26},c_{99},c_{72}$ by using the relationship in Equation (1), the coordinates of each cell is mapped to a numerical value of 25, 52, 89, 17. Also, chromosome 2 genes contains following cells: $c_{63},c_{29},c_{79},c_{95}$ is mapped to a numerical value of 26, 82, 87, 49. Also, the scope of both chromosomes is shown in Figure 10. The following is a series of mechanisms that modify the initial population to obtain optimal algorithm solutions.

### 4.5. *Parent Selection*

As mentioned before, μ was produced for the initial population, among which several chromosomes is selected to create the next generation by applying the intersection operators and mutations on them. Therefore, there are different methods to select including roulette wheel selection method. In this method, each chromosome is selected based on their suitability. In other words, the better the chromosome is, the more likely it is selected to produce the next generation. In this mentioned problem, each chromosome is assigned a numerical value based on the priority and the probability of selecting each chromosome as described in Equation (2). Then a random number is considered, and a chromosome is selected which is more optimal. Hence, μ is selected from the best chromosomes by selecting the roulette wheel as the first-generation population of the next generation.

(5) $$P\left(c_{1}\right)=\frac{Q(c_{1})}{\sum \left(Q\left(c_{1}\right)+Q\left(c_{2}\right)+\ldots +Q\left(c_{n}\right)\right)},$$

where the *P* function calculates the probability of selecting each chromosome, such as $c_{1}$ and the *Q* function obtains the superiority of each chromosome.

### 4.6. Crossover

**Figure 11:**
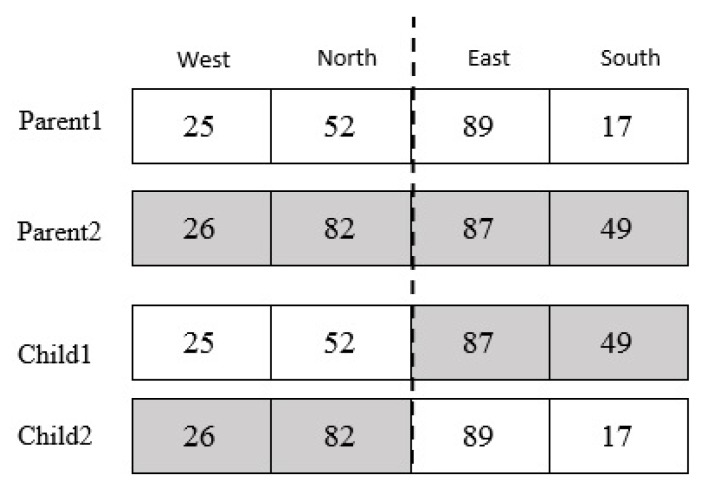
Product of child with crossover.

**Figure 12:**
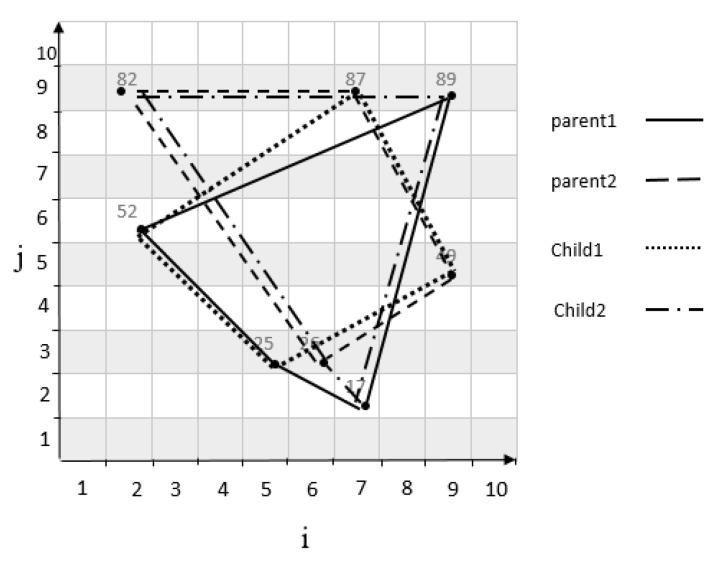
The scope of parent and offspring chromosomes.

After the parental selection, the single-point crossover operator is applied with a probability of 0.8. A point on parent chromosomes is picked randomly, and the genes from that point of the chromosomes are moved [20]. In this case, new offspring is created, which inherits the first child of the first, and second genes from the first parent, and the third and fourth genes from the second parent, and also the second child inherits first and second genes from the second parent, and the third and fourth genes from the first parent. It should be considered the limitations must be applied to a new child as well. For example, considering two parent chromosomes in Figure 9, and applying crossover operator on them, a new child is produced as shown in Figure 11. The movement scope of the parents and child’s chromosomes are shown in Figure 12.

### 4.7. Mutation

**Figure 13:**
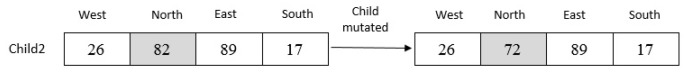
Mutation on second child.

**Figure 14:**
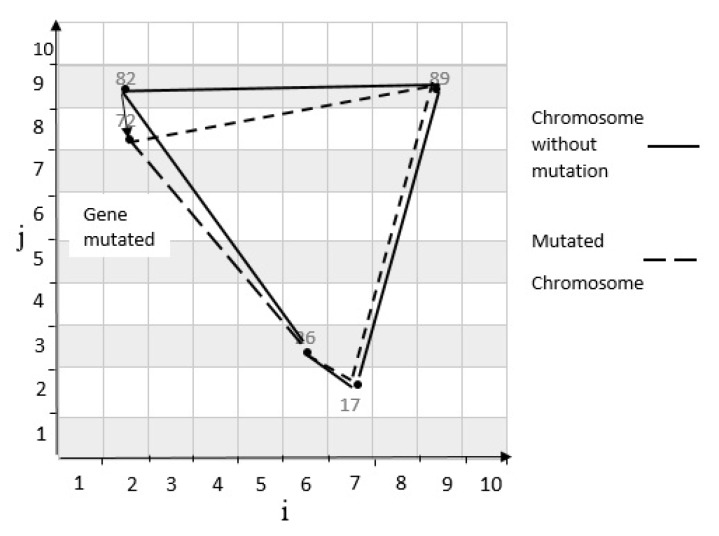
Mutated chromosome.

As mentioned in the previous subsections, a new child can be produced by using the crossover operator [21]. Now, if one of the genes of the child gets mutated, a new child will be produced. In this article, a random mutation method is from which a chromosome gene is randomly selected and replaced by a new amount. Also, the limitations mentioned above apply to new off-springs. For example, the mutation operator is applied (See Figure 13) to the second child as described in Figure 14. The mutation rate of the above problem is 0.1 and the mutation rate for the northern gene is 0.02. Therefore, the mutation rate of north gene is less than the mutation rate of the problem. Hence, the second child is mutated. The coordinates of the northern gene are changed to (2, 8) and the determinants are calculated as discussed next. The result of the determinism is positive, indicating that the new cell coordinates are properly selected, and they form a scope with other cells.

(6) $$R=\left|\begin{array}{cc} \left(i_{2}-i_{1}\right) & \left(i_{3}-i_{1}\right)\\ \left(j_{2}-j_{1}\right) & \left(j_{3}-j_{1}\right) \end{array}\right|=\left[\left(i_{2}-i_{1}\right)\times \left(j_{3}-j_{1}\right)-\left(i_{3}-i_{1}\right)\times \left(j_{2}-j_{1}\right)\right]$$

(6a) $$R=\left|\begin{array}{cc} \left(2-6\right) & \left(2-6\right)\\ \left(9-3\right) & \left(8-3\right) \end{array}\right|=4>0$$

(6b) $$C_{28}=\left(8-1\right)\times 10+2=72$$

### 4.8. Fitness Function

According to above, applying crossover and mutation on initial population, λ number chromosomes of the child was generated. Hence, μ chromosome from μ+λ chromosome should be selected as the next generation of population. Therefore, in order to select μ as the superior chromosome, they should be evaluated by fitness function based on problem’s objectives. The main objective of determining the optimum traffic zone is to find a zone with the least traffic load. Hence, the fitness function is defined based on a combination of two parameters of traffic load η and citizen satisfaction φ which is calculated as below:

(7) $$F\left(\eta ,\varphi \right)=\min\left(\frac{\eta }{\varphi }\right)$$

On the other hand, the fitness function must be displayed as a minimum or maximum, then the problem is considered to be the minimum for the purposes of a specific problem according to Equation (7).

#### 4.8.1. Traffic Load

Traffic load is one of the properties for evaluating each zone. Therefore, to calculate this property, the average traffic of all four cells should be obtained from the zone. Hence, the average traffic is obtained by dividing the total number of vehicles entered into each cell into the number of minutes within which the considered vehicles is in the given cell. Therefore, to calculate the number of vehicles per cell, assuming that each vehicle $u_{i}$ met cells of grid cell with m×m dimension in *t* time divided into one minute time-slot represented by $t=\left(1,2,3,4,\cdots ,T\right\}$ Therefore, the number of vehicles entering *t* in each cell is calculated according to Equation (8).

(8) $$N_{ij}\left(t\right)=\sum_{t=1}^{T}n_{t},\;i,j=1,2,\ldots ,m$$

In Equation (8), $N_{ij}\left(t\right)$ represents the total number of vehicles entering a cell with coordinates $\left(i,j\right)$ at time *t*.

After calculating the number of vehicles per cell, the average traffic $\tau_{ij}$ is obtained based on the total number of vehicles entering the specified number of minutes per cell, according to Equation (9).

(9) $$\tau_{ij}=\frac{N_{ij}\left(t\right)}{\sum t},\;i,j=1,2,3,\ldots ,m$$

In Equation (9), $N_{ij}\left(t\right)$ represents the total number of vehicles entering each cell with the coordinates $\left(i,j\right)$ and *t* is the total number of minutes the vehicle is in the desired cell.

Consequently, the traffic load η for each vehicle movement scope is calculated by dividing the total average traffic on the number of cells passing through vehicles $c_{ij}$ to the destination cell according to Equation (10).

(10) $$\eta =\frac{\sum \tau_{ij}}{\sum c_{ij}},\;i,j=1,2,3,\ldots ,m$$

#### 4.8.2. Citizen Satisfaction

Citizen satisfaction is introduced as another parameter of fitness function. It depends on the distance traveled by personal vehicles. In the limited traffic zone, only people who have permission to enter this zone can drive. Therefore, if individuals drive long distance in the is zone by private car, their satisfaction will increase. Otherwise, if the person does not have permission to enter the zone, he/she can only travel with personal vehicle near the zone. In such situation, a person who entered the limited traffic zone must park vehicles out of this zone and use the public transportation in limited traffic zone. In determining optimal traffic zone, satisfaction is equivalent to the number of cells from a grid cell with m×m dimensions, which can be passed by a personal vehicle.

(11) $$\varphi =\sum c_{ij},\;i,j=1,2,\ldots ,m$$

Once the satisfaction rate of the citizen is calculated, the traffic load and satisfaction rates are calculated with the fitness function. At first, the problem parameters are examined on the same scale, then normalization is performed as follows:

#### 4.8.3. Data Normalization

Data normalization means that when the data is not on a scale or domain, it needs to standardize on a scale. Therefore, normalization for determining the optimal traffic zone is needed due to different scale. Different methods have been proposed to normalize the data, but the method used in this study to normalize the data is defined as below:

(12) $$Z=\frac{\left(x-\overset{-}{x}\right)}{\sigma },$$

where *x* is random variable with average $\overset{-}{x}$ and Standard deviation σ [22]. The average $\left(x\right)$ is equal to the sum of the variables divided by *k* numbers which is calculated as below:

(13) $$\overset{-}{x}=\sum_{j=1}^{k}\frac{x_{j}}{k}$$

To calculate the standard deviation, the difference between each data and average data is obtained. Then divide to *k* numbers, and finally σ is calculated as follows:

(14) $$\sigma =\sqrt{\sum_{j=1}^{k}\frac{{\left(x_{j}-\overset{-}{x}\right)}^{2}}{k}}$$

After normalization, it will be used in the fitness function. The function is applied to each chromosome, thus fitness number to the μ initial population and λ child’s chromosome are assigned. Base on this number, the μ chromosome is selected from the chromosomes.

## 5. Experiments

This section uses a java-based simulator called The ONE [23] that models the movements of vehicles in a city. We compare ORDZ against OCP, RTZ, OEZ methods based on the two following scenarios: In the first scenario, we simulate the topology of the capital city of Iran, Tehran where ORDZ is compared with the two of the current restricted driving zones called RTZ and OEZ and a new method called OCP. In this scenario, the number of vehicles varies from 100,000 to 2,000,000 per day [24]. RTZ covers 32 km${}^{2}$ out of 730 km${}^{2}$ in which the vehicle’s mobility is limited from 6:30 a.m. to 5:00 p.m. on weekdays. OEZ covers 74 km${}^{2}$ that surrounds the RTZ zone [8]. The mobility model of vehicles is based on working day movement whereby 52% of vehicles are private with the maximum speed of 21 Km/h, 58% are cabs with the maximum speed of 21 km/h, and the rests are public transportation including trams and buses with the maximum speed of 15.6 km/h. The traffic and statistical information is collected from Statistical reports of the municipality of Tehran and Behruz et al. [24] represent the statistical information regarding the number of private and public vehicles in Tehran.

The second scenario considers a random topology of a city within the area of 10 km by 10 km. In this scenario, ORDZ, RTZ, OEZ and OCP methods are compared against each other under the random traffic distribution when the number of vehicles varies from 500,000 to 2,500,000 per day. The mobility model of vehicles is based on working day when travel destinations of people are put in the random zone. The speed of all cars is fixed and 50 Km/h. The length of time the cars move in this zone are considered the same.

### 5.1. First Scenario

**Figure 15:**
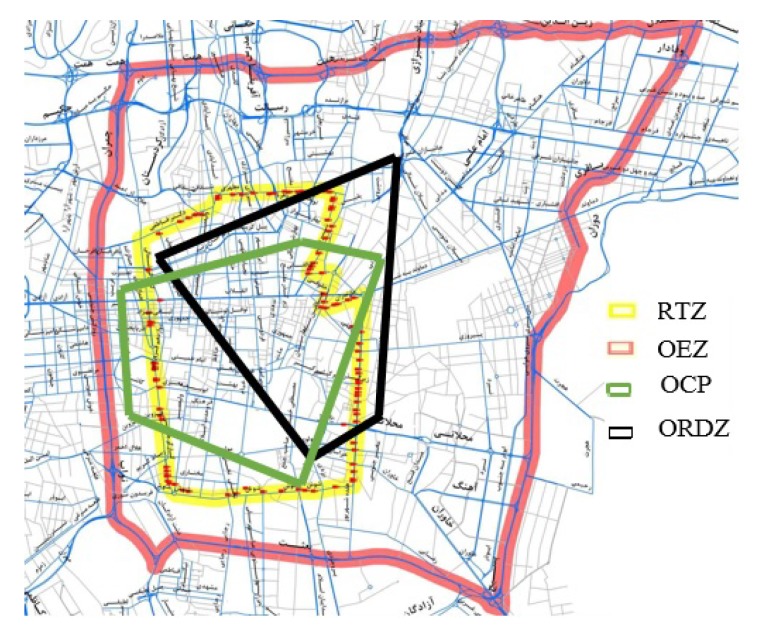
Restricted traffic zone of ORDZ, OCP, RTZ, OEZ methods.

Figure 15 illustrates the shape of ORDZ compared with RTZ, OEZ, and OCP in the city of Tehran, Iran. As shown in the figure, OEZ surrounds other zones and this is because the volume-to-capacity ratio in OEZ is more than other methods. Also, all four zones have a common area in terms of traffic load.

**Figure 16:**
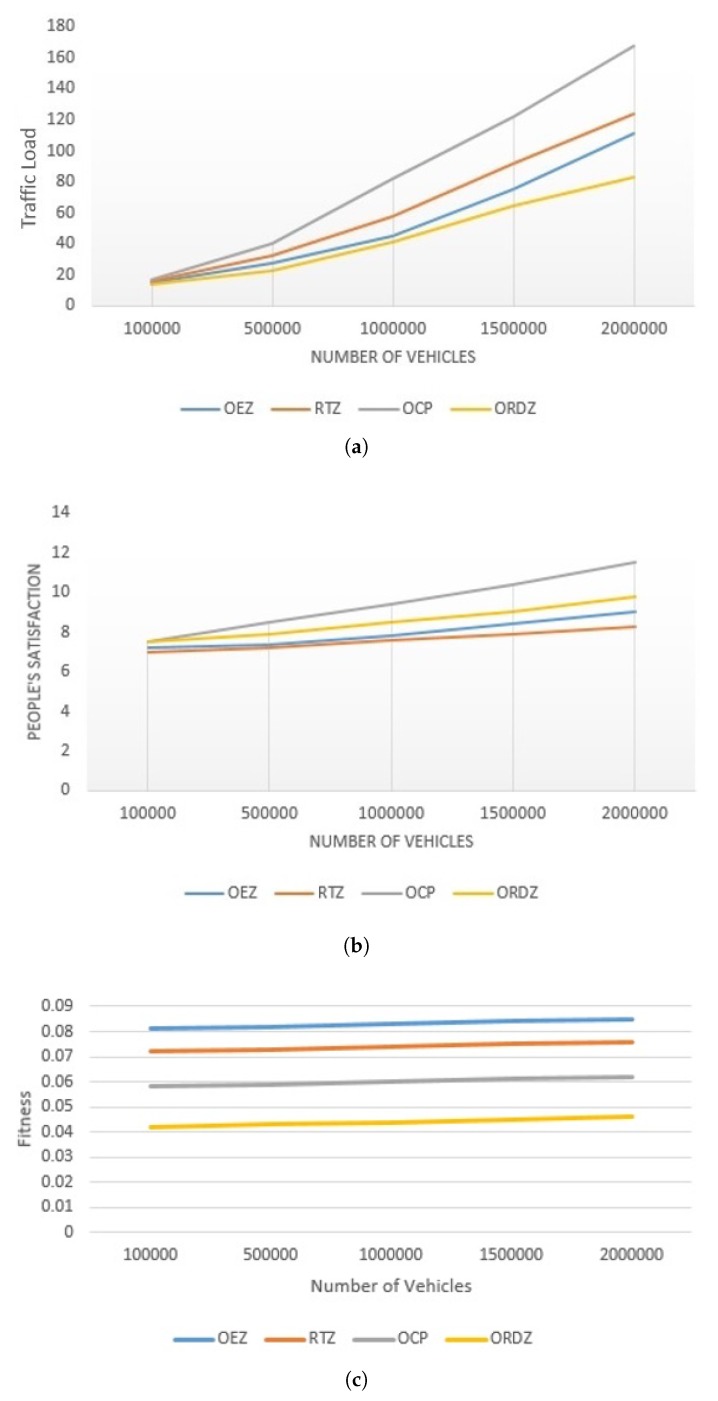
Comparison of different techniques based on: (**a**) impact of traffic load; (**b**) impact of citizen satisfaction; (**c**) fitness function for all methods.

Figure 16a compares ORDZ with OCP, RTZ and OEZ in terms of traffic load. As shown, ORDZ performs up to 23.81%, 19.87% and 14.23% better than OCP, OEZ and RTZ in terms of smoothing traffic. Please note that all of the above methods have same performance when vehicle density is low. According to the traffic flow theory [25], when the number of vehicles on the road is small then the traffic flow conditions are considered free. When the number of the vehicles per day is less than 100,000, all methods have same performance. On the other hand, when vehicle density reaches the maximum rate, the traffic flow change to unstable condition. This is because more than 100,000 vehicles per day are on the road with persistent stop and go driving patterns. We also find that the OCP and ORDZ have the most and least traffic load, respectively. In OCP, restricted traffic zone is determined by two factors which include travel cost and citizen satisfaction. In this method, the restricted traffic zone is determined based on the lowest travel cost and the highest satisfaction. However, in fact, the lowest tendency of people for using a toll zone show that in this method the advantage of reducing the traffic load is estimated much lower than considering the toll payment. On the other hand, the volume-to-capacity ratio of OEZ and RTZ are more than ORDZ. Therefore, it is expected that the traffic loads of these zones are more than ORDZ. It is clear that the performance of OCP, RTZ and OEZ methods in terms of traffic load are much lower than ORDZ.

Figure 16b, investigates the impact of car density on citizen satisfaction in different methods. The most and least satisfaction is accounted for OCP and RTZ methods. As shown in the figure, when the number of cars is low, the satisfaction of all methods is the same. This is because, according to the traffic flow theory, when the number of cars is low, the flow of traffic is smooth and the satisfaction of all the zones is similar. In contrast, when the number of vehicles increases, roads deal with extreme traffic load which varies in each restricted traffic zone. Therefore, it is predicted that the citizen satisfaction alters in all zones. As expected, the OCP method would have a better performance in gaining the citizen satisfaction than other methods, because this feature is a main factor in determining the restricted driving zone in OCP method. In other hand, ORDZ performs up to 9.23% and 7.57% better than OEZ and RTZ in terms of citizen satisfaction. This is because the restricted driving zone of RTZ and OEZ is wider than that of ORDZ Therefore, people should not drive with their private car in a larger area, so their dissatisfaction will increase. Although OCP has better performance than ORDZ in terms of citizen satisfaction, the impact of OCP in traffic reduction is less than ORDZ.

Figure 16c depicts trade-off between traffic load and citizen satisfaction and that is called fitness function. All methods which include RTZ, OEZ and OCP examine one metric instead of two, while ORDZ consider two metrics simultaneously. As is expected, ORDZ has the lowest fitness number about 4.43% and it introduces the best method for determining optimal traffic zone. While the OCP, RTZ and OEZ have less performance than ORDZ, it is nearly 5.68%, 7.34% and 8.75% respectively. This is because that RTZ and OEZ determine empirically restricted traffic zone in terms of traffic load and OCP just consider citizen satisfaction.

### 5.2. Second Scenario

**Figure 17:**
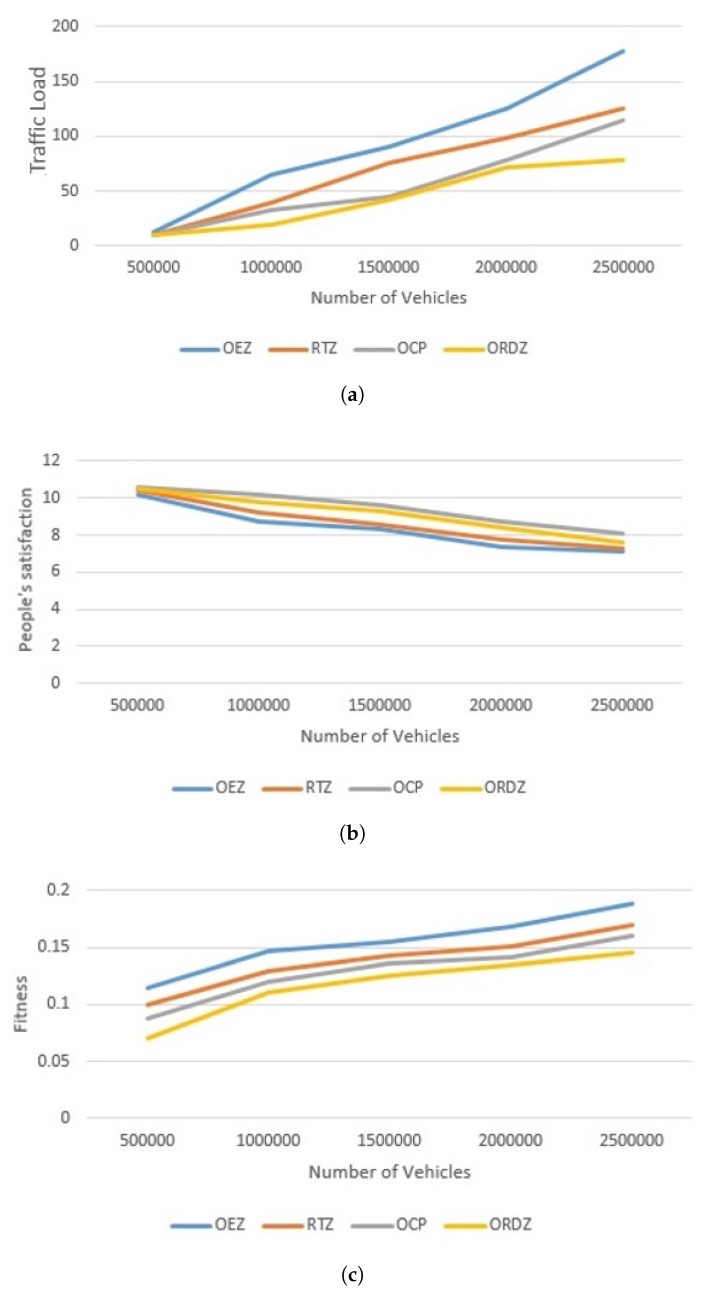
Comparison of different techniques based on: (**a**) impact of traffic load; (**b**) impact of citizen satisfaction; (**c**) compare fitness function for all methods.

In the second scenario, we consider the effects of the number of different vehicles moving into the city with a random topology. The model of vehicle movement is based on the workday movements [26]. This means the movement model of vehicles is predictable. The traffic load of our simulation in terms of traffic load is shown in Figure 17a. All of the above methods show similar performance when the vehicle density is low. When the number of vehicles on the road is less, the traffic flow conditions are considered free [25] and the traffic conditions will remain similar. As shown in Figure 17a, the lowest traffic load accounted for ORDZ (12%) and the highest for OEZ (27%). ORDZ has up to 7% improvement over OCP and up to 11% and 15% improvements over RTZ and OEZ, respectively. The RTZ and OEZ methods determine the traffic zone based on the previous data and experience as default, so when they are faced with the random movements of the vehicles, they show less performance. By contrast, since OCP method determines the traffic zone based on the random movement of the vehicles, it shows better performance than the other methods. This zone must have the lowest travel cost and the most citizen satisfaction, in which case such a small zone may be considered not reducing the traffic enough. Although OCP method has a significantly less traffic load than RTZ and OEZ, it has more traffic load than ORDZ.

In terms of citizen satisfaction, Figure 17b shows that the ORDZ method has achieved better citizen satisfaction than RTZ and OEZ. It has up to 12.23% and 16.36% improvement as compared to RTZ and OEZ, and down to 8.71% compared OCP. The traffic zone of RTZ and OEZ methods are predefined and it is not according to the random movements of the vehicles, so they have less satisfaction than the other methods. In contrast, although the traffic zone of both ORDZ and OCP methods are considered based on random movement of vehicles, the performance of OCP method shows more satisfaction than ORDZ. It is because that ORDZ traffic zone is almost according on random movement of vehicles, while OCP method determines the smaller zone to get the lowest travel cost, it will be more satisfying. However, the satisfaction of OCP method is more than that of ORDZ method, it shows a weaker performance in terms of traffic load.

Figure 17c depicts the trade-off between the traffic load and the satisfaction of the citizen, which is called the fitness function. The RTZ, OEZ and OCP methods just examine one metric, while the ORDZ considers two metrics simultaneously. As expected, ORDZ has the best performance in the random topology because this method determines the restricted driving zone based on the random movement of the vehicles with the least traffic load and the best citizen satisfaction. ORDZ shows the lowest fitness number at about 6.75, while the OCP, RTZ and OEZ show more fitness number to ORDZ, they are 18.47%, 26.24% and 36.31% respectively. This is because RTZ and OEZ empirically determine the restricted traffic zones and the OCP considers only the citizen satisfaction.

## 6. Conclusions

This paper proposes the ORDZ method to determine the optimal traffic zone with the least traffic load and the most satisfaction ratings. By default, the limited traffic zone was determined experimentally based on pollutant concentrations and traffic congestion. However, in the ORDZ method, the optimal traffic zone is determined by calculating the traffic load based on the number of vehicles per unit time in each zone and the calculation of citizen satisfaction from the proposed traffic zone based on the distances traveled to traffic zone. In this method, the chance of reaching the optimal zone is higher because of the randomness in the geographic locations of the limited traffic zone. A set of randomly selected locations creates a geometric shape that is in the scope of the proposed model. We consider the limits using the mathematical concepts such as determinism. As a result, the simulations of the system by OCP, RTZ and OEZ methods are compared with the changes in the number of vehicles in the scenario. Therefore, it can be stated that two parameters of traffic load and citizen satisfaction, due to the moderating role that they have in together, determine the optimal traffic zone. In this model, the traffic load is decreased, and citizen satisfaction increased. In the future work, it could be interesting to consider new factors such as the air pollution index as another factor in determining the optimal restricted driving zones. 

## Figures and Tables

**Table 1:** Comparison of traffic control methods.

Methods	Advantages	Disadvantages
Classic methods	• Reduce traffic volume • Reduce air pollution • Low cost	• Low quality • Low satisfaction • High travel time
Smart traffic lights methods	• Reduce delay time • Reduce traffic volume • Reduce air pollution	• High maintenance cost
Limited traffic zone methods	• Reduce traffic volume • Reduce air pollution • Low travel time	• Low satisfaction • Pay toll
Modern Technology Methods	• Reduce air pollution • Reduce Traffic volume • Environment friendly	• High maintenance cost

**Table 2:** Notations.

Notation	Description
m×m	Total number of grid cells
$\left(c_{11},\cdots ,c_{ij},\cdots ,c_{mm}\right\}\;i.j\in \left(1.2.\cdots .m\right\}$	Grid cells
$U=\left(u_{1},\cdots ,u_{i},\cdots ,u_{r}\right\}$	Vehicles
${MP}_{u_{i}}$	*i* -th vehicle movement pattern
$t=\left(1,2,3,\cdots ,T\right\}$	Discrete time
$\left(c_{ij},t\right)$	Couple-time vehicle movement pattern
*R*	Determinant of matrix
P(c1)	Probability of selection each chromosome
Q(c1)	Superiority of each chromosome
μ	Initial population
η	Traffic load
φ	citizen satisfaction rate
F(η,φ)	Fitness function
$N_{ij}\;\left(t\right)$	Total number of vehicles entering a cell
$\tau_{ij}$	Average traffic
*Z*	Data normalization
σ	Standard deviation

## References

[1] Barth M., Boriboonsomsin K. Real-World CO_2_ Impacts of Traffic Congestion. Proceedings of the 87th Annual Meeting of the Transportation Research Board.

[2] Chuang K.J., Chan C.C., Su T.C., Lee C.T., Tang C.S. (2007). The effect of urban air pollution on inflammation, oxidative stress, coagulation, and autonomic dysfunction in young adults. Am. J. Respir. Crit. Care Med..

[3] Chen L., Xu J., Zhang Q., Wang Q., Xue Y., Ren C. (2017). Evaluating impact of air pollution on different diseases in Shenzhen, China. IBM J. Res. Dev..

[4] John R.M., Francis F., Neelankavil J., Antony A., Devassy A., Jinesh K.J. Smart public transport system. Proceedings of the 2014 International Conference on Embedded Systems (ICES).

[5] Alam J., Manjo D.M. (2014). Advance traffic light system based on congestion estimation using fuzzy logic. Int. J. Emerg. Technol. Adv. Eng..

[6] Arora M., Banga V.K. Intelligent traffic light control system using morphological edge detection and fuzzy logic. Proceedings of the International Conference on Intelligent Computational Systems (ICICS’2012).

[7] Soret A., Guevara M., Baldasano J. (2014). The potential impacts of electric vehicles on air quality in the urban areas of Barcelona and Madrid (Spain). Atmos. Environ..

[8] Salarvandian F., Dijst M., Helbich M. (2017). Impact of traffic zones on mobility. J. Transp. Land Use.

[9] Eguiguren P. The 2008 Beijing Olympic Games: Spillover Effects on Air Quality and Health. Chicago Policy Review (Online). https://www.chicagopolicyreview.org.

[10] Ma H., He G. (2016). Effects of the post-olympics driving restrictions on air quality in Beijing. Sustainability.

[11] Chhatpar P., Doolani N., Shahani S., Priya R.L. Machine Learning Solutions to Vehicular Traffic Congestion. Proceedings of the 2018 International Conference on Smart City and Emerging Technology (ICSCET).

[12] Ma J., Huang X., Jiang Y. Applicable research of urban road traffic congestion based on rough set theory and Genetic Algorithm. Proceedings of the World Automation Congress 2012.

[13] Lambora A., Gupta K., Chopra K. Genetic Algorithm—A Literature Review. Proceedings of the 2019 International Conference on Machine Learning, Big Data, Cloud and Parallel Computing (COMITCon).

[14] Zhang X., Yang H. (2004). The optimal cordon-based network congestion pricing problem. Transp. Res. B Meth..

[15] Anwar A.M. (2009). Paradox between public transport and private car as a modal choice in policy formulation. J. Bangladesh Inst. Plan..

[16] Le T.P.L., Trinh T.A. (2016). Encouraging public transport use to reduce traffic congestion and air pollutant: A case study of Ho Chi Minh City. Procedia Eng..

[17] Geng Y., Cassandras C.G. (2015). Multi-intersection traffic light control with blocking. Discret. Event Dyn. Syst..

[18] Mehan S., Sharma V. Development of traffic light control system based on fuzzy logic. Proceedings of the International Conference on Advances in Computing and Artificial Intelligence.

[19] Ullah M.H., Gunawan T.S., Sharif M.R., Muhida R. Design of environmental friendly hybrid electric vehicle. Proceedings of the 2012 International Conference on Computer and Communication Engineering (ICCCE).

[20] Zhang Q., Chang S. An Improved Crossover Operator of Genetic Algorithm. Proceedings of the 2009 Second International Symposium on Computational Intelligence and Design.

[21] Lim S.M., Sultan A.B.M., Sulaiman M.N., Mustapha A., Leong K.Y. (2017). Crossover and mutation operators of genetic algorithms. Int. J. Mach. Learn. Cybern..

[22] Singh B.K., Verma K., Thoke A. (2015). Investigations on impact of feature normalization techniques on classifier’s performance in breast tumor classification. Int. J. Comput. Appl..

[23] Keränen A., Ott J., Kärkkäinen T. The ONE simulator for DTN protocol evaluation. Proceedings of the 2nd International Conference on Simulation Tools and Techniques.

[24] Behruz H., Safaie A., Chavoshy A.P. (2012). Tehran traffic congestion charging management: A success story. WIT Trans. Built Environ..

[25] Lieu H., Gartner N., Messer C., Rathi A. (1999). Traffic flow theory. Public Roads.

[26] Iranmanesh S., Chin K.W. (2015). A novel mobility-based routing protocol for semi-predictable disruption tolerant networks. Int. J. Wirel. Inf. Netw..

